# Orbital angular momentum detection device for vortex microwave photons

**DOI:** 10.1038/s44172-023-00056-5

**Published:** 2023-03-07

**Authors:** Chao Zhang, Xuefeng Jiang, Zheyuan Wang, Yuanhe Wang, Qiuli Wu, Xiangdong Xie, Wanyu Tian

**Affiliations:** Labs of Avionics, School of Aerospace Engineering, Tsinghua University, 100084 Beijing, PR China

**Keywords:** Electrical and electronic engineering, Electronic and spintronic devices

## Abstract

Orbital angular momentum (OAM), which was first discovered in the optical field, represents a new dimension of electromagnetic waves. However, the detection of OAM microwave photons, i.e., vortex microwave photons, at room temperature is difficult due to their low energy. Here we report a prototype of a vortex microwave photon detection device based on vortex electrons. Our OAM detection device efficiently distinguishes the intrinsic OAM in the microwave band, which is helpful for exploring new physical dimensions. In addition, the detection device can be enhanced with a vortex electron sorting device designed with electron holograms so that OAM microwave photon demultiplexing can be achieved. Finally, the OAM detection device has high practicability; i.e., not only it can be used at room temperature, but also it is much smaller than a particle accelerator system. To illustrate the significance of this method, we demonstrate an on-off keying transmission system based on our OAM detection device.

## Abstract (web summary)

Zhang and colleagues report an orbital angular momentum detection device for vortex microwave photons. Specifically, the device is able to distinguish the intrinsic orbital angular momentum mode of the vortex microwave photons with the diffraction pattern of the coupled vortex electrons at room temperature, occupying a much smaller area compared to a particle accelerator system. The device enables application of microwave photons in practical scenarios.

## Introduction

In the early twentieth century, Poynting derived the angular momentum of light in theory^1^. Experiments demonstrated that the polarization of light is the spin part of the angular momentum in 1936^2^. Allen et al. found that vortex Laguerre Gaussian light can transfer orbital angular momentum (OAM) to a suspended cylindrical lens^3^. Moreover, a verification experiment by mechanical torque, which means that the angular momentum (AM) contains not only the spin part but also the orbital part, was proposed. Afterward, OAM was also used in manipulation^4^, astronomy^5^, quantum information processing^6^, radar detection and imaging^7^, and wireless communications^8^.

The fact that an individual photon can carry OAM presents the most exciting practical possibilities for using OAM in the quantum domain^9^. However, only the characteristics of a large number of photons are utilized in most detection methods. For example, a single-photon with OAM can expand the coding dimension of a quantum key distribution or quantum direct communications, which can greatly improve the transmission rate^10,11^. However, for the aforementioned applications, diffraction and interference patterns are utilized to detect the OAM topological charge, such as diffraction gratings and Mach-Zehnder interferometers. Many individual photons are necessary to form a diffraction pattern in these methods. Comparatively, the detection of a single OAM photon cannot be completed in such methods. It was not until 2010 that Berkhout et al. separated the different OAM modes into different positions in space by some transformation optical lens^12^. Then, single-photon detectors were configured in different positions to detect a single-photon. The single-photon detectors, such as single-photon avalanche photodiodes, superconducting nanowire single-photon detectors, and quantum-dot field-effect transistor-based detectors^13^, have become increasingly mature in recent years.

Not only the optical beams but also the microwave OAM vortex beams also attract attentions from the academia^14,15^. The microwave OAM vortex beam can be generally radiated by the dedicated OAM antennas, but can also be generated by the uniform circular array (UCA) antennas. In 2007, Thidé et al. proposed that UCA antennas can be utilized to generate electro-magnetic (EM) wave beams with OAM in a low-frequency radio system, which paves the way for novel wireless communications concepts^14^. Compared with multiple-inputs and multiple-outputs (MIMO) communications based on a UCA, OAM communications based on UCA do not have many advantages except for line-of-sight transmission. The statistical OAM beams are synthesized by plane EM waves from different elements in the UCA with the well-defined vortex phase relationship, and the UCA emitted waves in the far field get interference, which forms the beam carrying the same OAM as the dedicated OAM antennas have. In this situation, OAM radio communications with vortex beams can be considered a subset of traditional MIMO communications^16,17^. The traditional antenna can be considered as a sensor of the electric field strength, which identifies the OAM of the beam with many microwave photons indirectly.

Besides the aforementioned vortex beam, the intrinsic OAM of single-photon can be transferred from electrons to microwave photons or vice versa directly. To utilize the new dimension efficiently from the intrinsic OAM of microwave photons, a dedicated OAM detection device for vortex microwave photons, which can directly measure the intrinsic OAM independent with the electric field strength, is necessary.

For the detection of OAM in the microwave band, most methods, such as the phase gradient method^18^, utilize the statistical characteristics of a large number of microwave photons. The reason is analyzed as the following. The energy of a single microwave photon is much lower than that of a single optical photon with OAM. In addition, the energy of a single microwave photon is much less than the background noise at room temperature. Hence, it is not easy to transmit a single microwave photon and display its quantum properties at room temperature and pressure. It was not until 2007 that Houck et al. completed the generation and detection of a single microwave photon in a low-temperature superconducting circuit^19^. In 2014, Andrews completed the transmission and manipulation of a single microwave photon by the bidirectional and efficient conversion between microwave and optical light in a low-temperature environment, i.e., below 4 K^20^.

In addition to EM waves with OAM, a few matter waves with OAM were also explored. For example, atomic vortices can be generated via the Raman process^21^. In addition, twisted neutron beams and vortex electron beams have been well investigated^22,23^. A vortex microwave photon can be generated by the energy level transition of vortex electrons. In 2014, the Project 8 working group of multiple European countries jointly completed the measurement of single-electron radiation^24^. It was verified that the EM wave energy of radiation follows discrete distribution characteristics, which verified the quantum properties of single-electron radiation. In addition, Sawant et al. found that relativistic electron beams in the cyclotron can generate microwave photons with high-order OAM^25^. In the same year, Katoh et al., from the Institute of Molecular Science in Japan, theoretically and experimentally proved that relativistic electrons in the cyclotron can also radiate microwave photons with OAM^26,27^. However, the facility is very cumbersome, e.g., the electron accelerator system tends to occupy an area greater than 1000 m^2^. Up to now, there is no OAM detection device for vortex microwave photons that works at room temperature. Moreover, for accelerating the research and development, the miniaturized facility for experiments is appreciated and expected.

In this paper, an intrinsic OAM detection device for vortex microwave photons is proposed. To show the effectiveness of the proposed vortex microwave photon detection device, vortex microwave photons are directly generated by the relativistic vortex electron. In the OAM detection device, the OAM of vortex microwave photons can be transferred to free electrons to generate vortex electrons. Afterward, diffraction patterns with a crystal thin film are used for the detection of vortex electrons with different OAM topological charges. The OAM carried by microwave photons can be analyzed by the different diffraction patterns with crystal thin films. There are three main advantages in the proposed OAM detection device: (1) The proposed OAM detection device can be utilized to distinguish OAM modes, i.e., the intrinsic OAM of microwave photons, which is very helpful for exploring and benefits from the new physical dimension of OAM; (2) the detection device can be enhanced with a vortex electron sorting device designed with the help of electron holograms so that OAM microwave photon demultiplexing can be achieved. Theoretically, even a single microwave photon can be identified if the sensitivity of one vortex electron is satisfied. (3) This OAM detection device for vortex microwave photons is highly practical, i.e., it can be used at room temperature and is much smaller than dilution refrigerators and particle accelerator systems. To validate the data transmission, a demonstration experiment is conducted based on on-off keying communications.

## Results

### Vortex microwave photon detection

OAM represents the rotational property of microwave photons. Furthermore, OAM can be divided into intrinsic OAM and extrinsic OAM^28–30^. Specifically, the intrinsic OAM reflects the internal rotation of the wave packet structure, while the extrinsic OAM depends on the spatial rotation of EM waves and varies with the change in the referred coordinate, e.g., the extrinsic OAM is expressed as ${{{{{{{{\bf{L}}}}}}}}}^{{{{{{{{\rm{ext}}}}}}}}}=\langle {{{{{{{\bf{r}}}}}}}}\rangle \times \langle {{{{{{{\bf{P}}}}}}}}\rangle$ ^28,30^, where **r** and **P** denote the vectors of the position and the linear momentum, respectively. To show that the quantum OAM is independent of the electric field strength, the physical dimensions of the electric field strength and OAM are evaluated. The physical dimensions of the electric field strength and OAM are **MLT**^−3^**I**^−1^ and **ML**^2^**T**^−1^, respectively, and are linearly independent^31^, where **M**, **L**, **T**, and **I** indicate the physical dimensions of the mass, length, time and electric current, respectively. Therefore, the new dimension besides electric field strength can be introduced based on the vortex microwave photons in wireless transmissions, and more degrees of freedom are available.

In recent studies, it has been demonstrated that the EM wave with OAM can be emitted and absorbed by the relativistic electrons in a magnetic field^32^. In the case of absorption, microwave photons of different frequencies and OAM modes can be used to drive vortex electrons with different Landau level quantum numbers. During the process of absorption, the energy, linear momentum and angular momentum are conserved^32^. Therefore, by detecting the OAM modes of vortex electrons, we can deduce the topological charges of vortex microwave photons. During this processing, we focus on the interaction of electrons with microwave photons. In other words, the particle characteristics of microwave photons with OAM are highlighted. The process of absorption cannot be easily explained by EM wave theory of traditional electro-dynamics, and quantum electro-dynamics (QED) should be employed. Based on QED, an OAM detection device for vortex microwave photons can be designed.

### Generation of vortex microwave photons

**Fig. 1:**
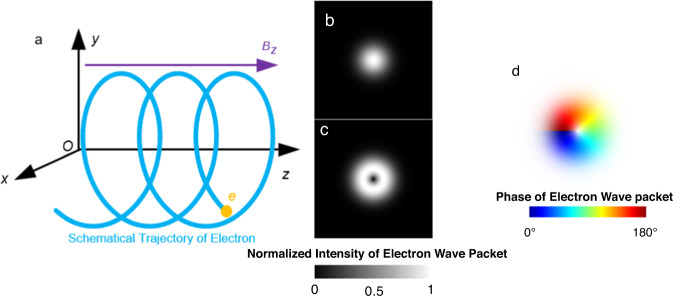
The generation mechanism of vortex microwave photons. **a** Electron rotation model in the generation of vortex microwave photons. An electron *e* rotates around the *z*-axis in the magnetic field **B** = (0, 0, *B*_*z*_), and the velocity of the electron is *v*. The blue line describes the trajectory of electron schematically. **b** The intensity of wave packet of electron in initial state of the Landau level, where the intensity is normalized by the maximized intensity. **c** The intensity of wave packet of electron in final level of the Landau state, where the intensity is normalized by the maximized intensity. **d** The generated vortex microwave photon.

As shown in Fig. 1, Landau levels can be formed when the electrons rotate in the longitudinal magnetic field. Vortex microwave photons can be generated when electrons transition from the high Landau level to the low Landau level. If gauge (+, −, −, −) is adopted and the natural unit is considered, the Dirac equation can be written as^32^:

1 $${{{{{{{\rm{i}}}}}}}}{\partial }_{t}\psi=[{{{{{{{\boldsymbol{\alpha }}}}}}}}\cdot (\hat{{{{{{{{\bf{p}}}}}}}}}+|{q}_{{{{{{{{\rm{e}}}}}}}}}|{{{{{{{\bf{A}}}}}}}})+{{{{{{{\boldsymbol{\beta }}}}}}}}{m}_{{{{{{{{\rm{e}}}}}}}}}+|{q}_{{{{{{{{\rm{e}}}}}}}}}|\varPhi ]\psi$$

where i is the imaginary unit, ${\partial }_{t}(\cdot )$ represents the derivative with respect to time, $\psi$ is the 4-component wavefunction, ${{{{{{{\boldsymbol{\alpha }}}}}}}}$ and ${{{{{{{\boldsymbol{\beta }}}}}}}}$ are the Dirac matrices, $\hat{{{{{{{{\bf{p}}}}}}}}}$ is the momentum operator, *m*_e_ is the mass of the electron, *q*_e_ is the charge of single-electron, and ${{{{{{{\bf{A}}}}}}}}$ and $\varPhi$ are the vector potential and the scalar potential, respectively. In addition, the magnetic induction is the curl of the vector potential, i.e., ${{{{{{{\bf{B}}}}}}}}=\nabla \times {{{{{{{\bf{A}}}}}}}}$. Therefore, the vector potential can be calculated by ${{{{{{{\bf{A}}}}}}}}=\frac{1}{2}{{{{{{{\bf{B}}}}}}}}\times {{{{{{{\bf{r}}}}}}}}=-\frac{1}{2}(Br\hat{\varphi })$, where ${{{{{{{\bf{r}}}}}}}}=(r,\varphi,z)$ represents the position vector in cylindrical system and the scalar potential$\varPhi=0$. According to Eq. (1), the energy of the relativistic electron is calculated by:

2 $$E=\sqrt{{m}_{{{{{{{{\rm{e}}}}}}}}}^{2}+{k}_{{{{{{{{\rm{z}}}}}}}}}^{2}+B|{q}_{{{{{{{{\rm{e}}}}}}}}}|(2n+m+|m |+1+2s)}$$

where *k*_*z*_ denotes the *z* component of the wavenumber, *n* is the radial quantum number, *m* is the magnetic quantum number, and *s* is the spin quantum number. Taking the Dirac positive energy electron wavefunction (up sign ↑ ) as an example, the wavefunction can be expressed as:

3 $${\psi }_{\uparrow }={{{{{{{{\rm{e}}}}}}}}}^{{{{{{{{\rm{i}}}}}}}}({k}_{z}z-Et+m\varphi )}{{{{{{{{\rm{e}}}}}}}}}^{-{\bar{r}}^{2}/2}\left\{{\bar{r}}^{m}{L}_{n}^{m}({\bar{r}}^{2})\left[\begin{array}{c}{E}_{+}\\ 0\\ {k}_{{{{{{{{\rm{z}}}}}}}}}\\ 0\end{array}\right]+{{{{{{{{\rm{ie}}}}}}}}}^{{{{{{{{\rm{i}}}}}}}}\varphi }{\bar{r}}^{m+1}{L}_{n}^{m+1}({\bar{r}}^{2})\left[\begin{array}{c}0\\ 0\\ 0\\ \sqrt{2B|{q}_{{{{{{{{\rm{e}}}}}}}}}|}\end{array}\right]\right\}$$

where $\bar{r}=\mu r$, $\mu=\sqrt{|{q}_{{{{{{{{\rm{e}}}}}}}}}B|/2}$, ${E}_{+}={m}_{{{{{{{{\rm{e}}}}}}}}}+E$, and ${L}_{n}^{m}(x)$ denotes the generalized Laguerre polynomial. It can be seen in Eq. (3) that the term ${{{{{{{{\rm{e}}}}}}}}}^{{{{{{{{\rm{i}}}}}}}}m\varphi }$ indicates the generation of vortex microwave photons with topological charge *m*.

After interaction with photon, i.e., radiating the vortex microwave photon, the magnetic quantum number of vortex electron changes from $m$ to $m^{\prime}$. The interaction process between the electron and a single-photon can be described by the transition matrix elements. Here the relation between the transition element and the angular momentum is concerned in proportion to $\delta (m-m^{\prime} -l\pm 1)$, where $\delta (\cdot )$ is the Dirac function, the $l$ is the OAM mode of vortex photon after interaction. The characteristics of Dirac function means that the angular momentum of radiated vortex photon is equal to the change of angular momentum of electron, i.e., $l\pm 1=m-m^{\prime}$, which indicates the angular momentum conservation during the generation of vortex photon^32^.

The vector potential ${A}_{k,l}$ of the single vortex photon with wave vector $k$ and the OAM mode $l$ radiated by the electron can be expressed as^32^:

4 $${A}_{k,l}={{{{{{{{\mathbf{\epsilon}}}}}}} }}\sqrt{\frac{1}{4{\pi }^{2}{\omega }_{0}}}{J}_{l}({k}_{\perp }r){a}_{k,l}{e}^{{{{{{{{\rm{i}}}}}}}}({k}_{{{{{{{\rm{P}}}}}}} }z+l\varphi )}+{{{{{{{{\mathbf{\epsilon}}}}}}} }}^{{{{\dagger}}} }\sqrt{\frac{1}{4{\pi }^{2}{\omega }_{0}}}{J}_{l}^{{{{\dagger}}} }({k}_{\perp }r){a}_{k,l}^{{{{\dagger}}} }{e}^{-{{{{{{{\rm{i}}}}}}}}({k}_{{{{{{{\rm{P}}}}}}} }z+l\varphi )},$$

where ${{{{{{{{\mathbf{\epsilon}}}}}}} }}$, ${k}_{\perp }$, ${k}_{{{{{{{\rm{P}}}}}}} }$ and ${J}_{l}(\cdot )$ denote the polarization vector, frequency of the vortex photon, transverse wave vector, propagation wave vector and $l$-order Bessel function of first kind, respectively. ${a}_{k,l}$ and ${a}_{k,l}^{{{{\dagger}}} }$ are annihilation and generation operator. ${(x)}^{{{{\dagger}}} }$returns the Hermitian conjugate of $x$. In Eq. (4), the term ${e}^{{{{{{{{\rm{i}}}}}}}}l\varphi }$ indicates the generation of the vortex microwave photon with topological charge $l$.

### Detection device structure and working principle

**Fig. 2:**
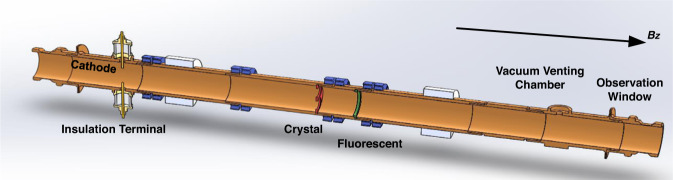
Orbital angular momentum (OAM) detection device for vortex microwave photons. In the proposed OAM detection device, the structure after the insulation terminal and before the crystal film can be regarded as a circular waveguide.

As shown in Fig. 2, the OAM detection device for vortex microwave photons is composed of an input window, an insulation terminal with a power supply, a cathode, a diffraction amplifier with a crystal, a fluorescent screen, an observation window and a vacuum venting chamber. The input and output windows are made of sapphire, which keeps the vortex microwave photon and vortex electron in a vacuum, i.e., usually lower than 5 × 10^−5^ Pa. The purpose of the vacuum is to ensure the efficiency of the electrons released from the cathode and to prevent the cathode from being oxidized. The electron can be released by the heated cathode at a high voltage, i.e., 1 kV. The longitudinal magnetic field aims to create a cyclotron motion for the electrons. In other words, the electrons can carry intrinsic OAM with the help of the longitudinal magnetic field.

The OAM quantum coupling module is utilized to couple the vortex microwave photon through the input window in free space to the relativistic electrons in the OAM detection device. Afterward, Landau levels can be formed by relativistic vortex electrons to absorb the vortex microwave photon. Because angular momentum satisfies the conservation law, the OAM of microwave photons can be completely transferred to the electrons, making the electrons become vortex electrons with intrinsic OAM^32^. Based on the wave-particle duality of relativistic electrons, the vortex electron beams can be diffracted with the help of the crystal made of gold foil to enlarge the observable transverse scale of the vortex electron beams. Then, the diffraction patterns can be mapped to the fluorescent screen and recorded by the camera through the observation window. Vortex electrons with different OAM modes can be distinguished according to different diffraction patterns. Based on the conservation law of angular momentum, the topological charges of vortex microwave photons can be deduced by distinguishing the different diffraction patterns. Then, if the initial state of the electrons is known (e.g., $m=0$ in the experiment), the total angular momentum of the microwave photons can be deduced by distinguishing the final state of the electrons. In addition, to measure the OAM, the polarization, i.e., the spin angular momentum, of the generated microwave photons must be known.

### Experimental scenario

**Fig. 3:**
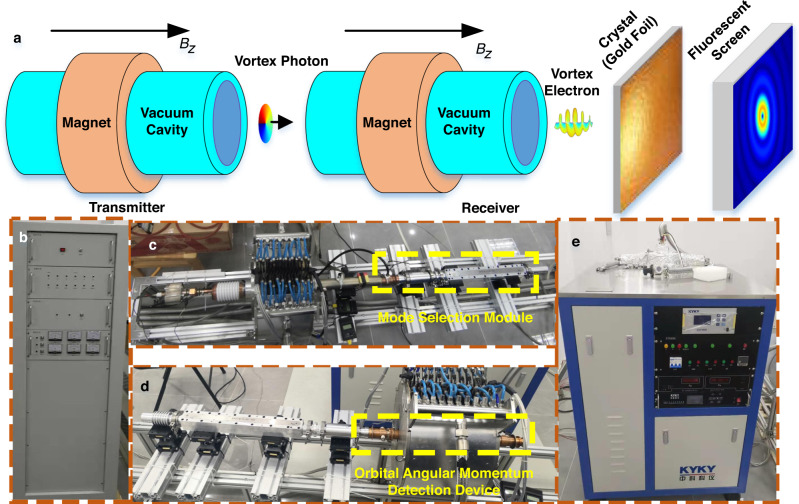
The experimental scenario of vortex microwave photon transmission. **a** Conceptual diagram of the vortex microwave photon transmission experiment comprising the transmitter and receiver. **b** High-voltage supply system, which provides the high voltage for the electron gun in the transmitter. **c** Generation of vortex microwave photons, where the orbital angular momentum (OAM) of the relativistic electron is transferred to the radiation microwave photons. Since the radiated OAM is determined by the harmonics^26,32^, the mode selection module, which is utilized to select the vortex microwave photons with the expected mode, is implemented with the resonant structure of cyclotron. **d** Detection of vortex microwave photons, where the OAM of the microwave photon is coupled to the vortex electron. **e** The vacuum pump provides a vacuum environment for the electron gun in the OAM detection device.

As shown in Fig. 3, the transmission experiment of vortex microwave photons is realized by mapping the OAM photons to vortex electrons. The cyclotron and the magnet device are configured, as shown in Fig. 3a. The corresponding electron cyclotron maser (ECM) is composed of an electron gun, high-voltage power, a magnetic field generator, a circular waveguide, and a mode selector module, which are shown from Fig. 3b–d. Moreover, the vacuum pump is shown in Fig. 3e.

**Fig. 4:**
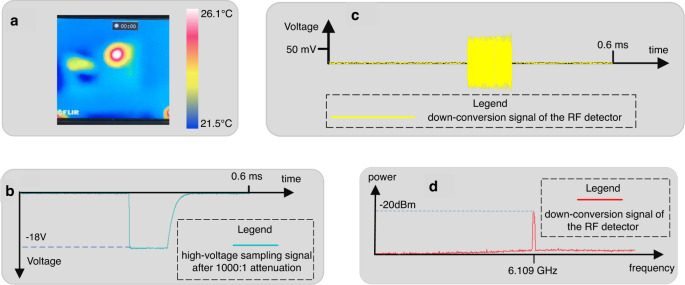
The experimental results of the generation of vortex microwave photons. **a** Thermal image of the radiator. The unit of the scale bar is Celsius, i.e., °C. **b** The high-voltage sampling signal (blue). **c** The down-conversion signal of the RF detector (yellow). **d** Radiation signal spectrum (red). The center and span of the frequency (*x*-axis) are 6 GHz and 1 GHz, respectively. Due to the range limit of the spectrum analyzer, frequency down-conversion with a 23 GHz local oscillator is adopted to obtain this spectrum. More information of the experiment results can be referred to Supplementary Fig. S4.

First, the radiation power is measured by adjusting the pulse width and repetition frequency of the high-voltage power supply. Due to the high radiation power, the thermal imager is used to observe the transmitted microwave beam emitted from the transmitter, as shown in Fig. 4a. Furthermore, the pulse waveform and the frequency can be checked by the coupling device and WR-28 waveguide with a Radio Frequency (RF) detector and oscilloscope. Two channels are utilized in the oscilloscope, as shown in Fig. 4b, c. The blue signal is the high-voltage sampling signal, which is regarded as the trigger signal. The yellow signal is the down-conversion signal of the RF detector, which corresponds to the high-voltage sampling signal. Figure 4d shows the spectrum measured by the spectrum analyzer. It can be found that the demand frequency, i.e., 29.1 GHz, is generated. In addition, the pulse waveform is also checked.

Note that it cannot be directly verified by Fig. 4 that vortex microwave photons are generated. Figure 4 can only show that the energy of the EM wave has been radiated from the cyclotron with the designed frequency. In addition, the intrinsic OAM of the vortex microwave photon cannot be identified by the energy and phase distribution of the radiated beam which occupies the statistically summation of the extrinsic OAM of the total photons in the beam. However, according to the diffraction pattern of the vortex electrons, the vortex microwave photons with a specific intrinsic OAM mode can be detected by the proposed OAM detection device.

**Fig. 5:**
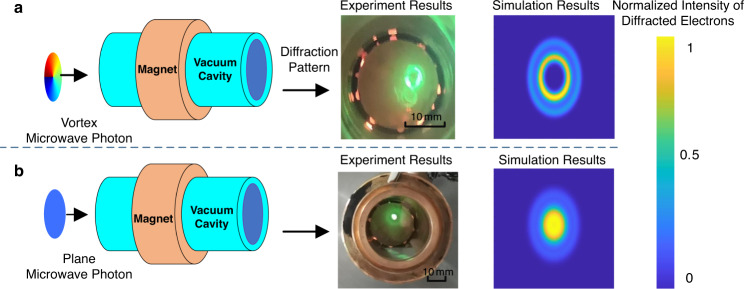
The experimental and simulation results of the vortex microwave photon detection device. **a** Experimental and simulation results of vortex microwave photons with OAM mode $l=1$. **b** Experimental and simulation results of plane microwave photons, i.e., the OAM mode $l=0$. The intensity legend is the normalization intensity, and the maximum intensity in the simulation results is normalized in the legend.

The picture of the vortex microwave photon detector is shown in Fig. 3d. The cathode (see Fig. 8 in “Method”) generates many electrons, and electrons are accelerated by the high voltage. The magnet device is used to generate a uniform magnetic field, which causes the electron to make a circular motion to absorb vortex microwave photons. The detection module consists of the gold foil and fluorescent screen, which aims to diffract the vortex electrons and display final diffraction patterns. The experimental and simulation results of the diffraction patterns with vortex microwave photons and plane microwave photons are illustrated in Fig. 5. In this paper, the initial state of the electrons is in $m=0$. When different OAM modes are transmitted, different diffraction patterns can be acquired by the fluorescent screen due to the conservation of the angular momentum during the absorption process of microwave photons.

The proposed OAM detection device for vortex microwave photons can only detect multiplexed microwave photons with different topological charge absolute values. $m$ is defined as the topological charge. The proposed OAM detection device can only detect OAM modes with different absolute values of $m$, i.e., $|m|$, because the diffraction patterns of $m=-1$ and $m=1$ are same.

In theory, the ideal diffraction pattern of vortex electrons with OAM is a doughnut shape. An elliptical shape of diffraction patterns appears in the experiments because the cathode releases electrons at an oblique angle in the proposed detection device. In addition, the intensity of the diffraction patterns fluctuates along an elliptical shape because only part of the electrons released by the cathode interact with the vortex microwave photons and then turn into vortex electrons. Vortex electrons with OAM interfere with the original electrons released by the cathode. In the experiment, the brightness of the diffraction pattern can be set by adjusting the current and voltage of the cathode. When there are microwave photons interacting with the electrons, not only the angular momentum but also the momentum and energy are transferred into the vortex electrons. Hence, the brightness of the diffraction pattern increases.

It is obvious that different diffraction patterns can be acquired when different vortex microwave photons are transmitted. In addition, the experimental results are in good agreement with the simulation results. Therefore, the proposed OAM detection device for vortex microwave photons can be utilized as a demodulator for an OAM shift keying (OAM-SK) transmission system. For example, vortex microwave photons are generated when the bit “1” is transmitted. Plane microwave photons are generated when the bit “0” is transmitted. In the receiver, the OAM modes can be distinguished by diffraction patterns, and the information can be recovered by the proposed OAM detection device. Due to the limit of the experimental conditions, only vortex microwave photons with OAM mode *l* = 1 are generated. To show the effectiveness of the proposed OAM detection device, the simulation results of high-order OAM are given in Supplementary Information Note S2 and Fig. S1.

### Demonstration of microwave photon transmission

**Fig. 6:**
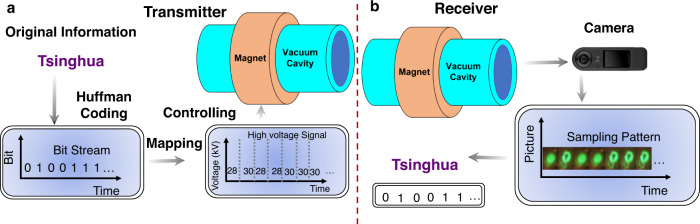
Wireless transmission experiment based on the proposed orbital angular momentum (OAM) detection device. **a** Block diagram of the transmission process, where the original information is directly encoded into the bit stream, and the transmitter is controlled to generate OAM microwave photons or plane microwave photons. **b** Block diagram of the receiving process, where the diffraction patterns collected by the camera are distinguished to recover the transmitted information.

To validate the aforementioned concepts and method, a detailed wireless communication system is built to perform the experiment of vortex microwave photon transmission in an indoor scenario, as shown in Fig. 6. We can rely on the vortex microwave photon generator prototype in ref. ^32^ and the detection method for vortex electrons in ref. ^33^.

During the experiment, the sequence “Tsinghua” in English is first encoded by the Huffman code and then transferred into the bit stream, for example, “010011…”. Subsequently, the high-voltage power supply is controlled according to the bit stream. In other words, the generation of electrons is controlled based on the bit stream. In the experiment, bit “0” denotes that the high-voltage power supply does not work, while bit “1” denotes that the high-voltage power supply works. In this situation, on-off keying of the OAM mode is realized by controlling the high-voltage power supply. Vortex microwave photons are generated when the bit “1” is transmitted. No vortex microwave photons but plane wave photons (OAM mode $l=0$) are generated by antenna when the bit “0” is transmitted.

At the receiver, OAM is transferred from the microwave photons to the vortex electrons based on the conservation of angular momentum. Hence, if the topological charges of the vortex electrons are detected correctly, the OAM mode of the microwave photons can be deduced. According to ref. ^33^, the diffraction pattern on the fluorescent screen can be used to distinguish vortex electrons with different topological charges. In the experiment, the main component of the fluorescent screen is the mixture of zinc sulfide (ZnS) and silver (Ag). Then, the bit stream can be demodulated according to the different diffraction patterns. Finally, the transmitted information, e.g., “Tsinghua”, can be recovered. The diffraction patterns are captured by the camera and converted into a digital signal. In the experiment, the demodulation of the bit stream is carried out on a personal computer to recover the transmitted information.

Certainly, the pilot sequence can be transmitted before the user data. In this situation, the diffraction pattern with vortex microwave photons (OAM mode $l\ne 0$) or plane microwave photons (OAM mode $l=0$) can be collected before data transmission to realize more efficient identification. Specifically, demodulation can be performed according to the pre-collected diffraction patterns. Due to the limited frame number per second of camera in the receiver, the encoding and decoding speeds of the system are 1 bit per second. In other words, the on-off switching of the diffraction patterns on the fluorescent screen is 1 Hz. Because of the indoor experiment, the transmission distance is 2 m. The details can be found in Supplementary Note S3, Figs. S2 and S3, and Supplementary Movie.

## Discussion

The radiation power of the current system can be estimated by calculating the temperature rise of the water load. In the current experiment, the radiation power is 20 W. By adjusting the transmission distance and the receiving angle, different receiving powers can be acquired. In the current experiment, a fluorescent screen and a camera are adopted to obtain the vortex electron diffraction pattern. The electric current of the vortex electrons must be large enough to make the brightness of the screen meet the threshold of the camera. This electric current of the vortex electrons is 1 μA. In addition, the input power of the vortex microwave photons is large enough to change the fluorescence pattern collected by the camera. The input power is 0.1 W, i.e., a large number of photons were used in the experiment.

The proposed OAM detection device for vortex microwave photons can be utilized to distinguish the OAM mode by different diffraction patterns. Hence, the OAM-SK transmission system can be realized by the proposed OAM detection device. However, for an OAM multiplexing transmission system, the proposed OAM detection device should be improved with a vortex electron sorter to map vortex electrons with different OAM modes to different positions in space. In this situation, if different OAM modes are transmitted in parallel with different bit streams, the information of each OAM mode can be recovered by sorting the vortex electrons with the corresponding OAM modes for OAM demultiplexing. Compared with the aforementioned OAM detection device, the coordinate transform and converging lens are supplemented with electron holograms^34^. In the coordinate transform module, the rectangular coordinate system is transformed into the log-polar coordinate system. Hence, the doughnut-shaped diffraction patterns are transformed into rectangular shapes with different phase gradients, which are determined by the OAM modes. Afterward, Fourier transformation is completed by the converge lens to conduct the vortex electrons with different OAM modes to different positions in space due to different phase gradients. Finally, the electron collectors can be deployed in the focus of the lens to obtain the electron with the corresponding OAM modes. Hence, different OAM modes can be separated and sorted, which is very helpful for OAM demultiplexing. However, compared with the OAM sorting device, i.e., electron holograms, the proposed OAM detection device is low cost and easy to implement. In other words, the proposed OAM detection device is easier to apply in engineering under current trechnical condition.

Actually, one vortex photon can only drive one electron to form one vortex electron. The diffraction pattern utilized in the current experiment is formed by the collection of many electrons. Hence, multiple electrons are necessary in this experiment. If there are multiple electrons and only one vortex photon is illuminated, only one electron can absorb the vortex photon and form the vortex electron. However, the other electrons remain unchanged. The vortex electron may form a doughnut-shaped pattern, and other electrons still form the traditional pattern. In this situation, the vortex electron is almost impossible to distinguish due to the severe interference from the other traditional electrons.

To realize single-photon detection, vortex electrons must converge to the separated points in the space domain according to the OAM modes rather than statistically forming diffraction patterns to avoid the aforementioned situation. With the help of the coordinate transform and converging lens in ref. ^31^, vortex electrons with the same topological charge can be conducted to the same position in space. In addition, vortex electrons with different OAM modes appear in different positions. Hence, we can distinguish the topological charge by estimating the location of the vortex electrons. Theoretically, even though there is only one photon, the final location of the corresponding sorted vortex electron can be determined with sufficient signal-to-noise ratio. In details, when the single vortex electron that is coupled with the single OAM microwave photon goes through electron sorter, the corresponding electron collector of the expected OAM mode can finally be obtained.

The proposed device directly detects vortex electrons and then detects microwave photons. The reason is that the energy of vortex electrons is much higher than that of microwave photons. In the current manuscript, the single microwave photon detection is not realized so far. Multiple photons are utilized so that the experiment can be successfully completed at room temperature. For the single microwave photon detection, to maintain the transformation efficiency between microwave photons and vortex electrons, the proposed device needs to work in a low-temperature environment to reduce the influence of thermal noise, e.g., the temperature needs to be below 1 K. Theoretically, if the sensitivity is satisfied, a single microwave photon can be identified. In addition, the original electrons, which do not interact with photons, can converge to the other electron collector. Hence, with the electron sorting device, the influence of the original electrons, which do not interact with photons, can be reduced.

Since only the intensity distribution information is utilized, some improvements can be made to the proposed OAM detection device. One of the possibilities is to utilize both the intensity and phase distribution information of vortex electrons in the receiver, e.g., an electron hologram^34^, which tends to identify the OAM mode with high accuracy, paves the way for real OAM sensors. This is a promising topic for future research.

## Conclusion

This paper provides an intrinsic OAM detection device for vortex microwave photons. To show the feasibility of the proposed OAM microwave detection device, a wireless transmission system is established. The system comprises a transmitting subsystem and a receiving subsystem. In the transmitter, relativistic electrons are utilized to generate vortex microwave photons with OAM. In the receiver, the electrons in the cyclotron can be used to couple the vortex microwave photons for obtaining the OAM. The modes of vortex microwave photons are detected by the diffraction of the corresponding vortex electrons. The above system and method can identify the intrinsic OAM mode of the microwave photons. Moreover, with the electron sorting module, the multiplexing of the vortex microwave photons can be achieved. The proposed detection device is more practical due to its small size (occupying an area less than 10 m^2^) and room temperature operation, which allow microwave photons to be applied in practical scenarios, and the new dimension characteristics of OAM can be thoroughly revealed.

## Method

### Design of OAM on-off keying transmission system

**Fig. 7:**
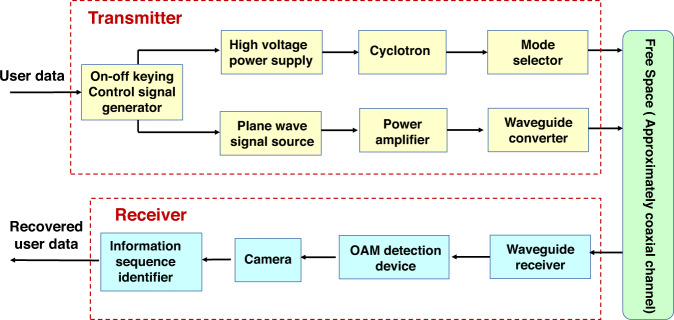
The schematic diagram of orbital angular momentum (OAM) on-off keying transmission system. The transmitter aims to radiate the wave with the switch of modes, where the plane wave signal generator and the cyclotron generate the waves with the mode 0 and 1 respectively. The receiver distinguishes the mode of the wave and recover the user data.

As shown in Fig. 7, the OAM on-off keying transmission system is composed of the transmitter and receiver. The function of each module can be described as Table 1. In the transmitter, the on-off keying control signal generator converts the user data, e.g., word “Tsinghua”, into the bit sequence with the bit rate 1 bit/s. The corresponding on-off keying control signals are generated for the high voltage power supply and the plane wave signal source respectively. Specifically, when the information bit is one, the high voltage power supply outputs 30 kV to cyclotron but the plane wave signal source does not work. When the information bit is zero, the plane wave signal source works and outputs the plane wave with 29.1 GHz, while the high voltage power supply outputs 28 kV to cyclotron. In our system, 28 kV is less than the threshold of the working voltage of cyclotron and vortex microwave photons are not generated by the cyclotron. Besides the high voltage, the longitudinal magnetic field with 5500 Gs and mode selector are necessary to make the cyclotron work at the second harmonic wave and select vortex microwave photons with mode 1. The length of the cyclotron is 1000 mm, and the diameter of the inwall is 32 mm. More details of the design of the cyclotron can be referred to ref. ^35^. The plane wave is generated by the plane wave signal source and enhanced by the power amplifier. The output port of the power amplifier is rectangular waveguide. In order to be consistent with the circular waveguide at the output of the mode selector, a rectangular waveguide to circular waveguide converter is used in the output of the power amplifier of the plane wave.

**Table 1:** The main functions of each module in orbital angular momentum (OAM) on-off keying transmission system.

Module name	Function
On-off keying control signal generator	Convert the user data into bit sequence, and select which branch works according to the bit 1 or 0.
High-voltage power supply	Provide the high voltage in the cyclotron.
Cyclotron	Generate the vortex microwave photons by the vortex electrons.
Mode selector	Select the vortex microwave photons with mode 1, then feed the plane wave into the waveguide combiner.
Plane wave signal source	Generate the sine signal for the plane wave.
Power amplifier	Enhance the energy of the plane wave.
Waveguide converter	Convert the plane wave from the rectangular to circular waveguide.
Waveguide receiver	Receive the electromagnetic wave from the free space.
OAM detection device	Transfer the OAM mode from the received microwave photons to the electrons, then detect the OAM mode by the diffraction pattern of the vortex electrons.
Camera	Record the diffraction pattern of vortex electrons on the fluorescent screen.
Information sequence identifier	Recover the user data by distinguishing the diffraction pattern recorded by the camera.

In the receiver, the transmitted wave is received by the waveguide receiver and enters the OAM detection device. The OAM detection device is implemented with cyclotron, and electron diffraction module. The total length is 584 mm and the diameter is 32 mm. As shown in Result, the plane wave (mode 0) and vortex microwave photon (mode 1) have different diffraction pattern in the OAM detection device, then the information sequence identifier can recover the bit by distinguishing the diffracted pattern in the fluorescent screen. To be specific, the camera can capture the diffraction pictures. By analyzing these pictures in the time domain, the user data, e.g., “Tsinghua”, can be recovered and decoded by information sequence identifier module. More specific information with regard to the OAM on-off keying transmission system can be referred to Supplementary Movie.

### OAM detection device design

The OAM detection device is the modified structure of the conventional Magnetron Injection Gun (MIG) with electrons and photons interaction, where the electron injection generated by the cathode along the axis of gyration exchanges the energy and angular momentum with the microwave photons carrying OAM. In the electromagnetic field, not only the energy of the microwave photon is absorbed by the electron, but also the OAM is transferred to the electron, which produces the vortex electrons with the corresponding OAM. Besides, the electron injection here can be regarded as a beam in the conventional MIG-generated electron injection. In our experiment, the high performance electron injection was obtained at an accelerating voltage of 1 kV, a current of 50 mA, a cathode inclination of 43° and a cathode surface height of 5.6 mm.

**Fig. 8:**
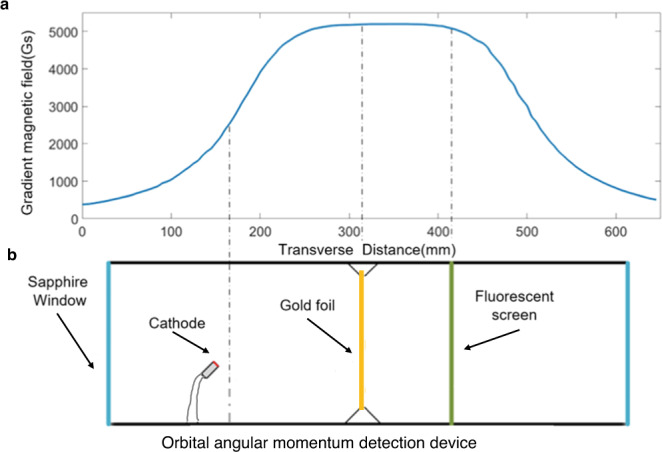
The schematic diagram of the magnetic field in the orbital angular momentum (OAM) detection device. **a** The transverse magnetic induction intensity distribution in the proposed OAM detection device. **b** The corresponding specific component in the detection device.

We designed and processed the energized solenoid magnet to measure the gradient magnetic field as shown in Fig. 8. There are two sapphire windows in the left and right ends of the OAM detection device for sealing the vacuum. Sapphire is commonly used as a raw material for electromagnetic wave lenses due to its high tensile stress and good dielectric property. To accurately sort out the OAM modes carried by the electrons, we set up a gold foil with thickness of 0.1 μm and diameter of 32 mm as a diffraction crystal in the OAM detection device. Then, the electrons carrying different OAM modes will show different patterns on the fluorescent screen after the diffraction with the gold foil.

The OAM detection module used in this work operates around the central frequency of *f*_c_ = 29.1 GHz and contains a circular waveguide to install the crystal diffraction thin film, i.e., the gold foil in the experiment, and the fluorescent screen more easily. The circular waveguide is designed with the help of electromagnetic computation commercial software, and the detection position in the magnet device is designed with the help of electric gun simulation software. A detailed analysis of the principle of the proposed detection device comprising the cathode can be found in Supplementary Note S1.

### Measurement setup

The experimental setup for the measurement of vortex microwave photons was established with the water load and coupling device. The water load aims to absorb the power of the radiated vortex microwave photons. Then, the radiation power can be estimated by calculating the temperature rise and the water flow rate of the water load. The coupling device aims to detect whether the vortex microwave photon is generated. It can couple the partial power of the radiation microwave photons to detect the frequency. If a high-voltage pulse is utilized, a rectangular pulse can also be observed with the help of the RF detector and oscilloscope. The radiation power is measured by adjusting the pulse width and repetition frequency of the high-voltage power supply. Two temperature sensors are installed at the inlet and outlet of the water load to monitor the temperature rise. In addition, the flow sensor is connected to the water load to measure the water flow. Based on the specific heat capacity formula, the radiation power can be measured. Besides the setup of measurement shown here, more specific information can be referred to Result and Supplementary Movie.

### Experimental setup of modulation and demodulation

As shown in Fig. 5a, OAM microwave photons with mode $l=1$ can be generated when the high voltage is 30 kV. There are no OAM microwave photons when the high voltage is 28 kV. In the on-off control signal generator, the user data are encoded into the bit stream to control the high voltage supply and the plane wave signal source respectively. Meanwhile, the probe (North Star PVM-5-2) with a ratio of 2000:1 is utilized to sample the voltage at 15 V and 14 V. Then, an analog subtractor is utilized to generate signals at 0 V and 1 V. Afterward, an inverter is used to generate the controlling signal for the plane wave signal source which produces the plane microwave photons. In other words, when vortex microwave photons with the intrinsic OAM are generated, the high voltage is 30 kV, and the controlling signal for plane microwave photons is 0 V. When the high voltage is 28 kV, and the controlling signal for plane microwave photons is 1 V, only plane microwave photons can be generated.

In the on-off keying transmission experiment, the pilot sequence can be transmitted before the user data. In this situation, the diffraction patterns corresponding to the OAM microwave photons and the plane microwave photons can be collected before the data transmission, so that the effect of the pattern distortion because of the imperfect transmission can be overcome. Specifically, the demodulation can be performed according to the pre-collected diffraction patterns. Due to the limited frame number per second of the camera in the receiver, the transmission rate is configured as one symbol per second. Hence, the renewal rate of the diffraction patterns in the fluorescent screen is 1 Hz. To get more descriptions with regard to the modulation and demodulation schemes, Supplementary Note S3 and Supplementary Movie can offer more helpful information. Furthermore, the QED analysis of microwaves with the quantum OAM and the statistical OAM can be found in ref. ^36^, so that the readers can know more details about the difference between the vortex microwave photon and the statistical vortex microwave beam.

### Supplementary information

Supplementary Information

Description of Additional Supplementary Files

Supplementary Movie 1

## Data Availability

The data that support the findings of this study are available from the corresponding author upon request.
