# Supplementary material for: Orbital angular momentum detection device for vortex microwave photons
Source: Commun Eng. 2023 Mar 7;2:11. doi: 10.1038/s44172-023-00056-5 (PMC10955893; doi:10.1038/s44172-023-00056-5)
Supplement: Supplementary file 1 — Supplementary Information [file 44172_2023_56_MOESM1_ESM.pdf]

Supplementary Material for

Orbital Angular Momentum Detection Device for Vortex Microwave Photons

Chao Zhang*, Xuefeng Jiang, Zheyuan Wang, Yuanhe Wang, Qiuli Wu, Xiangdong Xie, and Wanyu Tian
Labs of Avionics, School of Aerospace Engineering, Tsinghua University, Beijing, 100084, P. R. China
Corresponding Author: Chao Zhang, E-mail: zhangchao@tsinghua.edu.cn

THIS PDF FILE INCLUDES:

- Supplementary Note S1. Principle of OAM Detection Device
- Supplementary Notes S2. Diffraction Pattern of Vortex Electrons with Crystal
- Supplementary Notes S3. OAM On-Off Keying Demonstration
- Supplementary Fig. S1. Phase fronts and 3D views of diffraction patterns with different OAM modes including pure OAM modes and superposition modes.
- Supplementary Fig. S2. Diffraction patterns corresponding to Huffman codes.
- Supplementary Fig. S3. The screenshot of demodulation GUI in the host computer.
- Supplementary Fig. S4. The screenshots of the experimental results of the generation of vortex microwave photons.
- Supplementary Reference.

Supplementary Notes S1. Principle of OAM Detection Device

The mathematical model and principle of the OAM detection device consist of two main parts: 1) the interaction between microwave photons with intrinsic OAM and relativistic vortex electrons; 2) the diffraction of the vortex electrons in the OAM detection device. Here the brief conclusions are given, more details with respect to the physical derivation can be referred to our previously published paper references [S1] and [S2].

In the first part, the OAM transition probability between microwave photons and vortex electrons is derived theoretically. Usually, the transition probability is low when the OAM transition between microwave photons and vortex electrons occurs in free space. However, the situation is different when the relativistic vortex electrons move in a magnetic field. Relativistic electrons form Landau levels in a magnetic field. When transitions between these levels occur, it can be proved that the EM wave with OAM can be emitted and absorbed by the relativistic electron. This provides the possibility to detect the intrinsic OAM of vortex electrons to analyze the intrinsic OAM of the vortex microwave photons[S1]. The analysis of OAM transfer principles is also described in "Detection device structure and working principle" in the main body of the article.

In the second part, we propose theoretically that the diffraction pattern of the polycrystal thin film is more suitable to be used for the detection of the vortex electron with superposed OAM modes in the magnetic field[S2]. Specifically, we derive the wave functions of Laguerre-Gaussian (LG) vortex electrons based on the Schrödinger-Pauli equation and, as a result, analyze the diffraction of vortex electrons with individual atoms. According to the different distributions of atoms in different crystals, the total diffractions with the single crystal and polycrystal can be calculated for both single and superposed OAM modes. Our results show that polycrystal diffraction can be used to detect the different superposed OAM electron beams. In the case of the coordinate transformation, the theoretical detection rate of OAM mode is very high, i.e., even close to 100%[S2]. Because of the discernibility at the centimeter scale, these polycrystal diffraction mathematical models provide the capability to identify and demultiplex the superposed OAM modes on the macroscopic scale for our detection and wireless transmission experiments in "Experimental scenario" and "Demonstration of microwave photon transmission" in the main body of the article.

Supplementary Notes S2. Diffraction Pattern of Vortex Electrons with Crystal

Due to the limit of experimental condition, only vortex microwave photons with OAM mode l = 1 are generated in the experiment. To show the effectiveness of the proposed OAM detection device, the simulation results of high order OAM are given as follows. The material of the diffraction crystal thin film is gold. For pure OAM modes, as the topological charge of the OAM mode increases, the central

cavity of the diffraction pattern increases. The cell orientation of polycrystals is distributed in almost all directions. Hence, the pre-diffraction beam shape is mostly preserved and only the diffraction pattern is amplified, which is similar to Fraunhofer diffraction of free space OAM electron beams. It is concluded in the following Fig. S1 that the diffraction patterns are different for different OAM modes, which reveals feasibility of the proposed method to detect the OAM microwave photons.

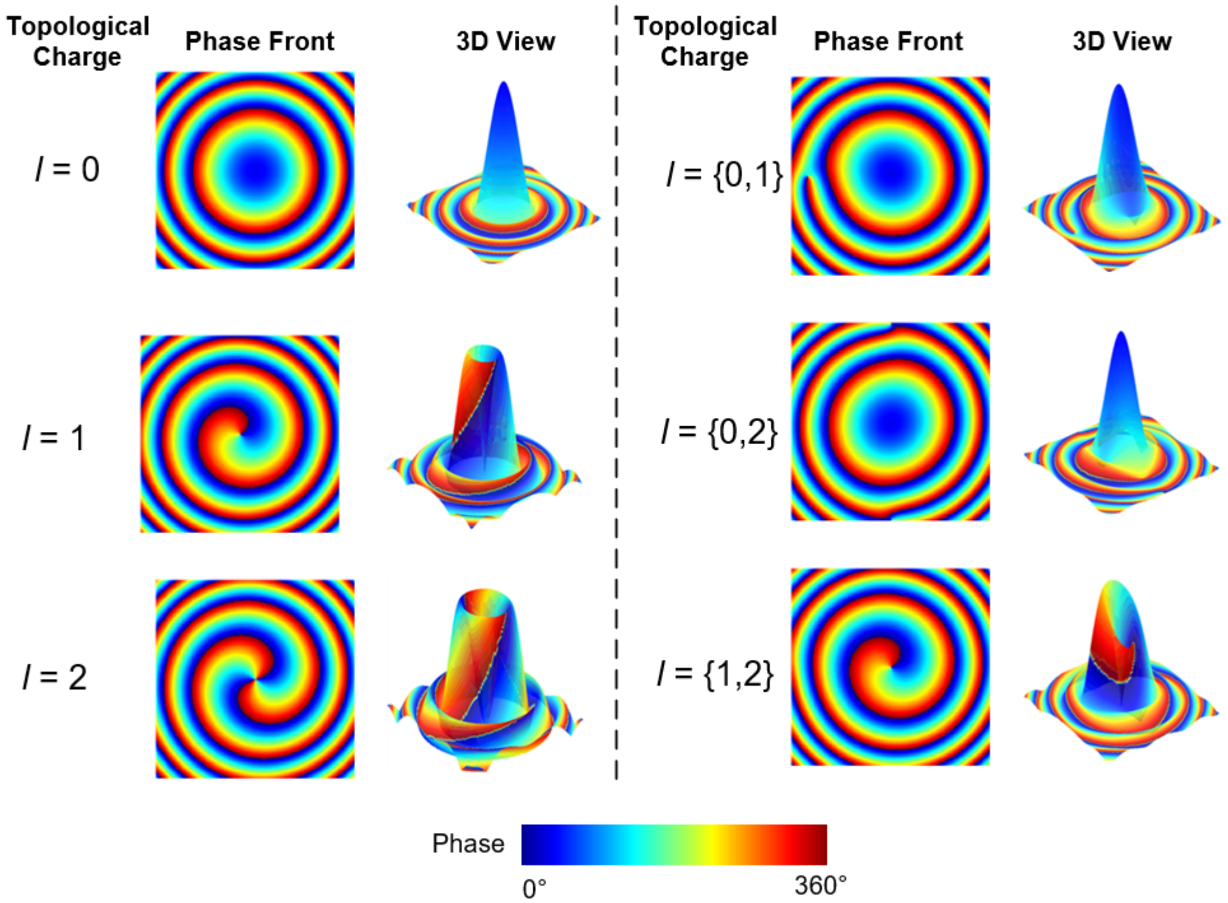

Supplementary Fig. S1. Phase fronts and 3D views of diffraction patterns with different OAM modes including pure OAM modes and superposition modes. The left panel shows the pure OAM modes, and the right panel shows the OAM superposition modes, and color denotes phase of wave.

Supplementary Notes S3. OAM On-Off Keying Demonstration

Figure S2 denotes the diffraction patterns corresponding to different Huffman Codes. The following Fig. S3 shows the Graphical User Interface (GUI) of the data recovery system in the computer.

The detailed demonstration experiment with the proposed OAM detection device can be referred to the uploaded video in Supplementary Information. Furthermore, the original screenshots of the experimental results of the generation of vortex microwave photons are shown in Fig. S4, which can be utilized as the reference for Fig. 4 in the main article.

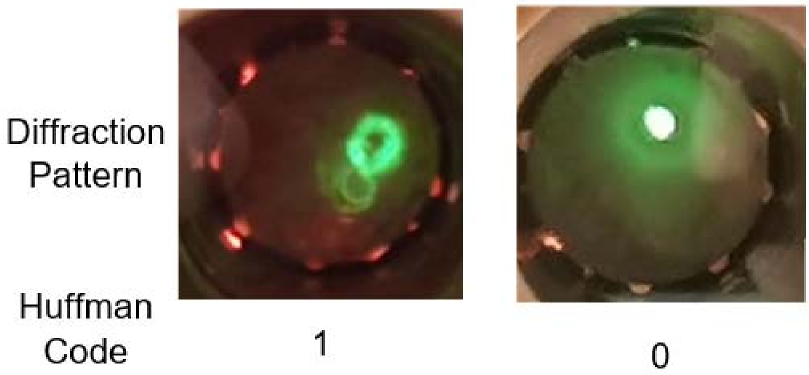

Supplementary Fig. S2. Diffraction patterns corresponding to Huffman codes. When diffraction pattern is in the shape of doughnut, it means that the mode 1 is transmitted and the corresponding code is 1, which is shown in the left, otherwise the mode 0 is transmitted.

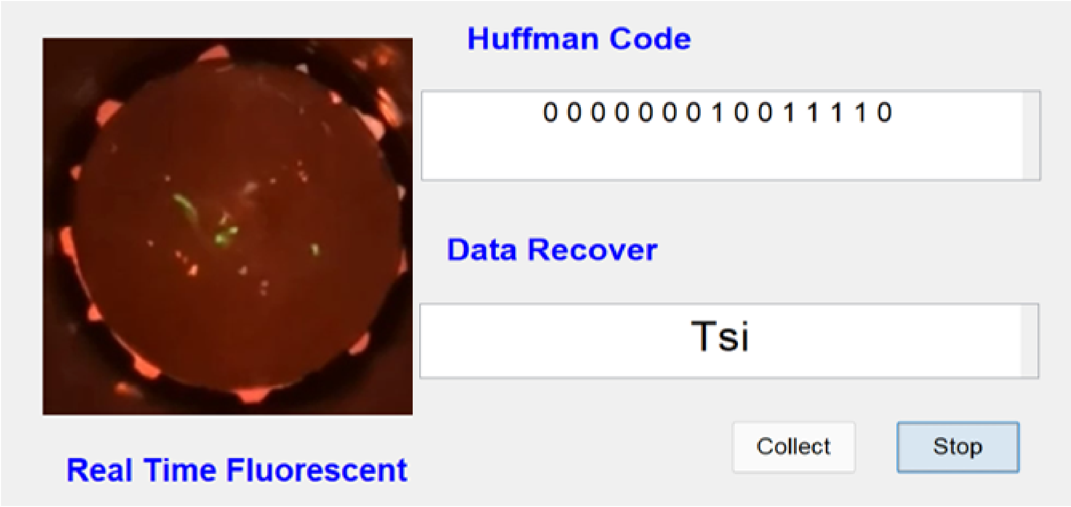

Supplementary Fig. S3. The screenshots of demodulation GUI in the host computer. The photograph at the left gives real time fluorescent screen, the top right shows the code detected by the fluorescent screen, the recovered data are given at bottom right. It should be noted that in the experiment, the sequence with multiple bit "0"s is transmitted as the pilot sequence before the data.

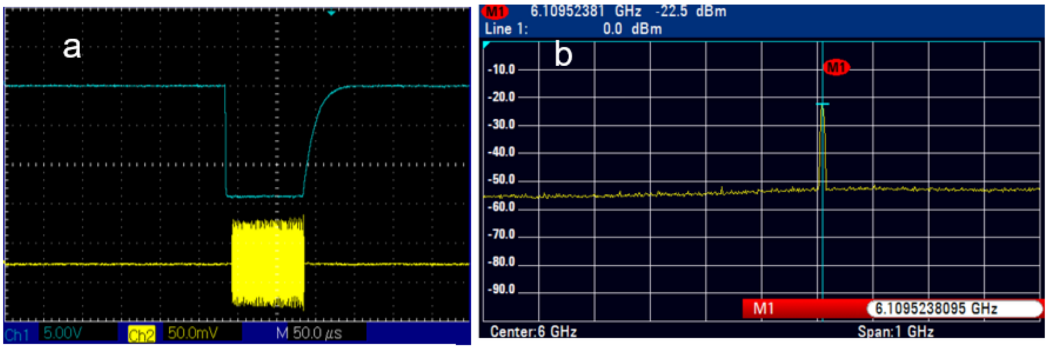

Supplementary Fig. S4. The screenshot of the experimental results of the generation of vortex microwave photons. a, The high-voltage sampling signal (the blue channel). **b,** the down-conversion signal of the RF detector (the yellow channel). The resolution of the x-axis is 50 μs. The resolution of the y-axis for the blue channel and the yellow channel are 5 V and 50 mV, respectively.

Supplementary References

[S1] Zhang C, Xu P, Jiang X. Vortex electron generated by microwave photon with orbital angular momentum in a magnetic field. *AIP Advances*, **10**, 105230 (2020).

[S2] Zhang C, Xu P, Jiang X. Detecting superposed orbital angular momentum states in the magnetic field by the crystal diffraction. *Eur. Phys. J. Plus*, **136**, 60 (2021).
