# Supplementary material for: Orbital angular momentum detection device for vortex microwave photons
Source: Commun Eng. 2023 Mar 7;2:11. doi: 10.1038/s44172-023-00056-5 (PMC10955893; doi:10.1038/s44172-023-00056-5)
Supplement: Supplementary file 2 — Description of Additional Supplementary Files [file 44172_2023_56_MOESM2_ESM.pdf]

Description of Additional Supplementary Files

File name: Supplementary Movie 1

Description:

Title: Experiment of OAM wireless transmission with the OAM detection device

Legend: The video records the experiment conducted with the proposed OAM detection device, which shows the details of the experimental setup and results of OAM mode key shift wireless transmission.
